# RIP140 Represses the “Brown-in-White” Adipocyte Program Including a Futile Cycle of Triacyclglycerol Breakdown and Synthesis

**DOI:** 10.1210/me.2013-1254

**Published:** 2014-01-30

**Authors:** Evangelos Kiskinis, Lemonia Chatzeli, Edward Curry, Myrsini Kaforou, Andrea Frontini, Saverio Cinti, Giovanni Montana, Malcolm G. Parker, Mark Christian

**Affiliations:** Department of Stem Cell and Regenerative Biology (E.K.), Harvard Stem Cell Institute, Harvard University, Cambridge, Massachusetts 02138; Institute of Reproductive and Developmental Biology (L.C., E.C., M.G.P.), Faculty of Medicine, Imperial College London, W12 0NN, United Kingdom; Department of Mathematics (M.K., G.M.), Statistics Section, Imperial College London, London SW7 2AZ, United Kingdom; Department of Experimental and Clinical Medicine (A.F., S.C.), University of Ancona, (Politecnica delle Marche), 60126 Ancona, Italy; Division of Metabolic and Vascular Health (M.C.), Warwick Medical School, University of Warwick, Coventry, CV4 7AL, United Kingdom

## Abstract

Receptor-interacting protein 140 (RIP140) is a corepressor of nuclear receptors that is highly expressed in adipose tissues. We investigated the role of RIP140 in conditionally immortal preadipocyte cell lines prepared from white or brown fat depots. In white adipocytes, a large set of brown fat-associated genes was up-regulated in the absence of RIP140. In contrast, a relatively minor role can be ascribed to RIP140 in the control of basal gene expression in differentiated brown adipocytes because significant changes were observed only in Ptgds and Fabp3. The minor role of RIP140 in brown adipocytes correlates with the similar histology and uncoupling protein 1 and CIDEA staining in knockout compared with wild-type brown adipose tissue (BAT). In contrast, RIP140 knockout sc white adipose tissue (WAT) shows increased numbers of multilocular adipocytes with elevated staining for uncoupling protein 1 and CIDEA. Furthermore in a white adipocyte cell line, the markers of BRITE adipocytes, Tbx1, CD137, Tmem26, Cited1, and Epsti1 were repressed in the presence of RIP140 as was Prdm16. Microarray analysis of wild-type and RIP140-knockout white fat revealed elevated expression of genes associated with cold-induced expression or high expression in BAT. A set of genes associated with a futile cycle of triacylglycerol breakdown and resynthesis and functional assays revealed that glycerol kinase and glycerol-3-phosphate dehydrogenase activity as well as [^3^H]glycerol incorporation were elevated in the absence of RIP140. Thus, RIP140 blocks the BRITE program in WAT, preventing the expression of brown fat genes and inhibiting a triacylglycerol futile cycle, with important implications for energy homeostasis.

There are two types of adipose tissue with opposing functions: white adipose tissue (WAT) is associated with dynamic energy storage, whereas brown adipose tissue (BAT) is associated with energy expenditure (1). White and brown adipocytes are often interspersed within the same depot, and their relative amount determines the color of the depot (2). In mice, BAT is found mainly in the interscapular depot whereas WAT is predominant in all other depots. However, cellular composition can change depending on the genetic background, sex, age, environmental temperature, and nutritional status (3). Chronic exposure to cold, treatment with β3-adrenergic agonist, or overfeeding induces the development of brown-like adipocytes (beige or BRITE adipocytes) within WAT, mainly in subcutaneous (sc) depots (4). Although originally thought to be present only in neonates, functional BAT has recently been identified in adult humans (5). Because the thermogenic activity of BAT mass is associated with protection against overnutrition and glucose intolerance (6) it is important to characterize the regulators of the BAT-specific program.

Receptor-interacting protein 140 (RIP140) is a nuclear receptor (NR) coregulator highly expressed in metabolic and reproductive tissues (7). The physiological role of RIP140 was indicated by the phenotype of RIP140-null mice, which are lean with a 70% reduction in total body fat mass and have higher oxygen consumption (8, 9). When challenged with high-fat diet RIP140-knockout mice are resistant to obesity and have increased insulin sensitivity and glucose tolerance. They are also resistant to age- and diet-induced hepatic steatosis, indicating that they do not use alternative fat depots. Importantly, RIP140-null mice have elevated expression of Ucp1 and carnitine palmitoyltransferase 1b (Cpt1b) in WAT, indicating that the lean phenotype is due to altered energy balance (8).

RIP140 was first identified as a cofactor that is recruited to the estrogen receptor in presence of 17β-estradiol (10). Subsequently, it was shown to interact with nearly all the NRs and despite its ligand-dependent recruitment, it usually acts to suppress their activity (7). In WAT and muscle, RIP140 interacts with key NRs such as peroxisome proliferator-activated receptors (PPARs), thyroid hormone receptors, and estrogen-related receptor (ERR)α to suppress catabolic signaling pathways. Expression analysis of differentiated adipocytes from RIP140-null mouse embryo fibroblasts and from 3T3-L1 adipocytes depleted of RIP140, showed the up-regulation of clusters of genes that are highly expressed in BAT, including genes that are involved in mitochondrial biogenesis and function, energy dissipation, and catabolic pathways (11, 12). Because many of these genes are targets of PPAR coactivator (PGC)-1α it seems that RIP140 and PGC-1α play mutually opposing roles.

In BAT, the role of RIP140 has not been fully elucidated. However, its importance is implicated in newborn RIP140-null mice that have reduced BAT mass, lower core body temperature, and altered BAT gene expression (13). In brown adipocytes, RIP140 is recruited to the Cidea promoter through ERRα and nuclear respiratory factor-1 (14). In addition, RIP140 can suppress Ucp1 expression through liver X receptor-α in brown adipocytes by antagonizing the binding of PPARγ/PGC-1α to the Ucp1 promoter (15).

Due to the critical role of RIP140 in the control of energy homeostasis, elucidating its role in the regulation of WAT and BAT activity might provide new therapeutic targets for obesity and diabetes. Here, we have found that RIP140 has only a minor role in gene regulation in brown adipocytes compared with its function in white adipocytes, where it repressed genes associated with energy utilization. Furthermore, a group of genes associated with a futile cycle of TAG breakdown and resynthesis is controlled by RIP140, highlighting an additional mechanism for heat generation in adipose tissues.

## Materials and Methods

### Microarray analysis of gene expression

Total RNA was isolated from 3 samples each of RIPKO-1 and RIPKO-L cells after adipocyte differentiation (10 days). Two RIP140-lentivirus-transduced cell lines were used for profiling in order to avoid variation due to differences in the sites of incorporation of the virus. For adipose tissue analysis, RNA was extracted from sc WAT of wild-type and RIP140^−/−^ 3-month-old male mice maintained on standard chow. Mice used in this study were backcrossed 8 generations to C57BL/6J background. All animal studies were carried out according to UK Home Office guidelines.

Affymetrix array hybridization and scanning were performed by the CSC/IC Microarray Centre, Imperial College London, Hammersmith Campus, using Affymetrix MOE 430 v2.0 GeneChips. For the array on RNA from the cell lines, data were analyzed with d-CHIP software (16). The microarray data are available at http://www.ebi.ac.uk/arrayexpress/ under accession number E-MIMR-42.

For the array of wild-type and RIP140-knockout WAT array, background correction, probe-level summarization and normalization of raw data from Affymetrix CEL files were performed across the full set of samples using Robust Multichip Average (17) as implemented in the Affy package of Bioconductor. Genes with a significant difference in diet-based induction between the wild-type and knock-out cell lines were identified using LIMMA (18) to calculate empirical Bayes moderated *t* statistics from a linear model evaluating the effect of diet in each cell line. The Benjamini-Hochberg approach to multiple testing adjustment was used to select a list of genes with a family-wise error rate of 0.05. *P* values from the moderated *t* statistics were used to rank the genes in terms of the significance of the difference between diet-induced change in expression between the wild-type and knock-out cell lines.

For temperature and fat depot-specific expression, groups of four 10-week-old female 129Sv mice (Charles River Laboratories) were housed separately and kept at 22°C. Subsequently, one group of mice was placed at 28°C and one group was placed at 6°C for 10 days. Animals were housed and culled as previously reported (19) and adipose tissues (interscapular BAT and sc, mesenteric, and gonadal WAT) were collected. Gene expression microarray was performed by Almac diagnostics UK using GeneChip Mouse Genome 430A 2.0 Array (Affymetrix) on RNA extracted from interscapular BAT and sc and mesenteric WAT from mice exposed to 28°C or 6°C. Three biological replicates were used. The analysis was conducted using R programming language and Bioconductor (20). After background adjustment, summarization, and normalization of the raw intensities, the data were analyzed using affy (21) and limma (22). Pair-wise comparisons between conditions were performed. A linear model was fitted to the expression values of each probe, modified to account for the replicated samples and assuming no interaction between the genes. The moderated *t* statistic was computed for each probe and for each contrast providing *P* values that were corrected for multiple testing using the Benjamini-Hochberg method to control the false discovery rate (23). Probes with adjusted *P* values <.01 were considered statistically significant.

### Light microscopy and immunohistochemistry

Serial paraffin sections, 3 μm in thickness, were obtained from each specimen. Some were stained with hematoxylin and eosin to assess morphology; the others were used for immunohistochemistry. After dewaxing, antigen retrieval was achieved with a pressure cooker treatment (90°C for 20 minutes) by soaking sections in a sodium citrate buffer 0.01 M, pH 6.0. After a thorough rinse in PBS, sections were reacted with 0.3% H_2_O_2_ (in PBS; 30 minutes) to block endogenous peroxidase, rinsed with PBS, and incubated in a 3% blocking solution (in PBS; 60 minutes). Then they were incubated with the primary antibodies (in PBS; overnight at 4°C). After a thorough rinse in PBS, sections were incubated in a 1:200 (vol/vol) biotinylated secondary antibody solution in PBS for 30 minutes (goat antirabbit IgG; Vector Laboratories). Histochemical reactions were performed using Vectastain ABC kit (Vector Laboratories) and Sigma Fast 3,3 ′-diaminobenzidine (Sigma-Aldrich) as the substrate. Sections were finally counterstained with hematoxylin, dehydrated, and mounted in Entellan. The following primary antibodies were used on serial sections: rabbit anti-uncoupling protein 1 (UCP1) (Abcam; catalog no. 10983 at diluition 1:600 [vol/vol]) and rabbit anti-Cidea (Sigma-Aldrich; catalog no. C7977 at diluition 1:100 [vol/vol]). Staining was never observed when the primary antibody was omitted. Tissue sections were observed with a Nikon Eclipse E800 light microscope (Nikon Instruments) using different objectives, and digital images were captured with a Nikon DXM 1220 camera.

### Generation of conditionally immortalized preadipocyte cell lines

Primary cultures were prepared from sc WAT and interscapular depots of RIP140^+/−^ and RIP140^−/−^ mice as described previously (24). Preadipocytes were immortalized by retroviral-mediated expression of temperature-sensitive SV40 large T-antigen H-2kb-tsA58 (a gift from P. Jat). Cells were cultured at 33°C and selected with puromycin (5 μg/mL). To differentiate into adipocytes, they were grown to 90% confluence and then cultured at 37°C. They were exposed, 48 hours after confluence, to differentiation medium consisting of DMEM:F12 supplemented with 10% FBS, insulin (1 μg/mL), dexamethasone (250 nM), isobutylmethylxanthine (0.5 mM), and rosiglitazone (2.5 nM). After 48 hours, the medium was replaced with fresh medium supplemented with 10% FBS and insulin and cultured for 7–10 days.

### Oil Red O staining

Oil Red O stain was used to confirm the presence of lipid in cells. Cells were washed with PBS, fixed in 2% paraformaldehyde and 0.2% glutaraldehyde in PBS for 15 minutes, and then rinsed with PBS. They were then stained with Oil Red O (in isopropanol) for 20 minutes and rinsed in 60% isopropanol and then PBS.

### Quantitative real-time PCR (Q-RT-PCR)

RNA extraction was performed with Trizol reagent (Invitrogen Life Technologies) according to manufacturer's guidelines. RNA (1 μg) was used for cDNA synthesis according to the manufacturer's protocols using the Moloney murine leukemia virus reverse transcriptase (Sigma) with random hexamers (Invitrogen) after treatment with deoxyribonuclease (Sigma). Synthesized cDNA was then used for Q-RT-PCR using SYBR Green I (Life Technologies) according to manufacturer's instructions. Thermocycling was performed on an ABI PRISM 7700 Sequence Detection System (Life Technologies). Fold changes were calculated by the ΔΔCt method using L19 as a housekeeping gene (25). PCR primers used in this study are listed in Supplemental Table 1 published on The Endocrine Society's Journals Online web site at http://mend.endojournals.org.

### Reporter gene assay

Reporter assays were carried out in 96-well plates with 20 ng of reporter gene and 5 ng of pRL-CMV, in the absence or presence of PPARγ (5 ng) or pEF-RIP140, using jetPRIME transfection reagent (Polyplus transfection) to transiently transfect 293 cells. The Gyk luciferase promoter construct (−2009/+57 bp) (26) was provided by M. Lazar. Luciferase activity was measure using Steadylite Plus (PerkinElmer) and normalized to *Renilla* activity.

### Chromatin immunoprecipitation assay

Chromatin immunoprecipitation assays were performed as described previously (27). Briefly, 3T3-L1 adipocytes were fixed with the protein-protein cross-linking reagent dimethyl adipimidate 2 HCl (Pierce Chemical Co.) at 10 mM in DMEM for 30 minutes followed by 1% formaldehyde in PBS for 10 minutes at 37°C. Harvested cells were then lysed, sonicated, and immunoprecipitated with Protein A/G PLUS-agarose (Santa Cruz Biotechnology, Inc.) according to the manufacturer's instructions using the following antibodies: RIP140 (a kind gift from Hongwu Chen), PPARγ (sc-7196; Santa Cruz Biotechnology), and thyroid hormone receptor-associated protein 220/MED1 (sc-5334; Santa Cruz). DNA fragments were purified with QIAquick PCR purification kit (QIAGEN) and enrichment relative to input was determined by quantitative PCR. Primer sequences were taken from Reference 26.

### Glycerol kinase enzymatic activity assay

Glycerol kinase (GYK) activity assay was performed as previously described (28) with minor modifications. Adipocytes were differentiated until day 8 in 10-cm diameter culture dishes, scraped on ice in 0.5 mL of glycerol kinase extraction buffer, and freeze/thawed in liquid nitrogen to allow cell lysis. Cell lysates were then centrifuged at 12 000 rpm for 15 minutes at 40°C, and protein concentration of the supernatant was determined by the Bradford assay. Protein (10 μg) was then incubated with 50 μL assay buffer that included 5 mM ATP, 4 mM glycerol, and 500 μM [^3^H]glycerol. The reaction was incubated at 37°C, for 150 minutes and terminated with the addition of 100 μL ethanol-methanol (97:3). [^3^H]glycerol was phosphorylated to [^3^H]glycerol-3-phosphate-dehydrogenase according to the relative activity of GYK in each of the samples. Fifty microliters of the reaction was then spotted onto DE-81 Whatman filters (Whatman) which were air dried, washed in water under a vacuum 5 times, and air-dried. Radioactivity adhering to the filters was measured by liquid scintillation counting in 10 mL of scintillant, corresponding to relative GYK activity.

### Glycerol incorporation assay

Glycerol incorporation was adapted from previously published protocols (28, 29) with minor modifications. Adipocytes were differentiated until day 8 in 10-cm diameter culture dishes serum starved for 3 hours in Kreb's Ringer phosphate buffer followed by a switch to normal differentiation medium (DMEM with 10% FBS and insulin), supplemented with 5 mM glucose, 100 μM glycerol, and 25 μCi [^3^H]glycerol and incubated for an additional 2 hours at 37°C. The amount of labeled glycerol incorporated into fat is directly proportional to the endogenous GYK activity in metabolically active adipocytes. Total fat fractions were isolated after cells were vigorously washed 3 times in ice-cold PBS, collected with scraping in 0.25 mL of 0.2M NaCl and freeze/thawed in liquid nitrogen. Once cells were lysed, 750 μL of chloroform-methanol (2:1) was added to the thawed cell suspension after which the mixture was vortex mixed and centrifuged for 5 minutes at 6000 × *g*. The top aqueous fraction was discarded and the bottom lipid layer washed once with 1 mL of methanol-chloroform-water (48:3:47). [^3^H]Glycerol incorporated into these fat fractions was quantified by liquid scintillation counting in vials containing 10 mL of scintillant.

### Glycerol-3-phosphate-dehydrogenase enzymatic activity assay

Glycerol-3-phosphate dehydrogenase (GPDH) activity was evaluated using the GPDH activity assay kit (Takara; catalog no. MK246) according to manufacturer's instructions from RIPKO-1 and RIPKO-L cells. Briefly, adipocytes were differentiated until day 8 in 10-cm diameter culture dishes; a protein extract was prepared using the kit's enzyme extraction buffer and equal amounts of protein for each sample in serial dilutions were then incubated with substrate buffer containing dihydroxyacetone phosphate. The reaction was followed by monitoring the absorbance at 1-minute intervals for 10 minutes. The relative GPDH activity was calculated according to the suggested formula of the kit.

### Triglyceride assay

Total cellular triglyceride levels in RIPKO-1 and RIPKO-L cells were determined with the triglyceride colorimetric assay kit (Cayman Chemical) according to the manufacturer's instructions. Data was normalized to DNA content measured using fluorescent DNA quantitation kit (Bio-Rad Laboratories).

### Western blot

Protein levels of glycerol kinase and glycerol-3-phosphate dehydrogenase were determined as described previously (27) using the following antibodies: GYK (Abcam, ab20428) and GPDH1 (Abcam, ab34492).

## Results

**Figure 1.**
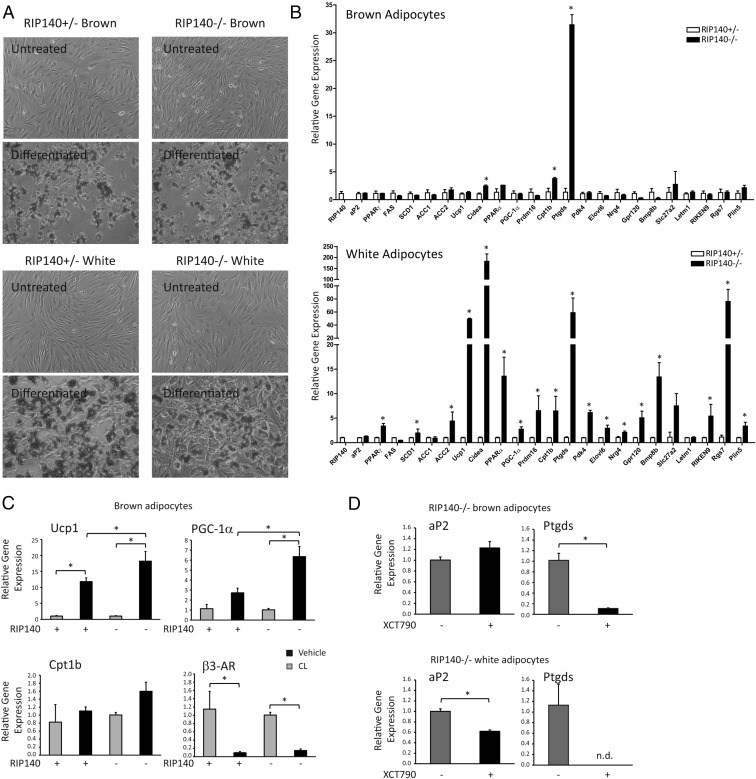
RIP140 repressed BAT genes in white adipocytes with only a minor role in brown adipocytes. A, Oil Red O stain of undifferentiated and differentiated conditionally immortal preadipocyte cell lines derived from sc WAT and interscapular BAT from RIP140^+/−^ and ^−/−^ mice. B, The expression of adipocyte genes in differentiated cells derived from brown (upper panel) or white fat (lower panel) was determined by Q-RT-PCR and expressed relative to RIP140^+/−^ cells. *, *P* < .05, Student's *t* test for comparison of RIP140^+/−^ vs ^−/−^ cells for expression of individual genes, performed separately for brown and white cells. C, The expression of Ucp1, Ctp1b, PGC-1α, and β3-AR was monitored in differentiated brown adipocytes knockout (−) or heterozygous (+) for RIP140 in the absence and presence of 10 μM CL 316,234 (CL) treatment for 5 hours. *, *P* < .05, ANOVA with Bonferroni's correction for differences in individual gene expression in RIP140-knockout and heterozygous cells, in the absence and presence of CL. D, aP2 and Ptgds expression was determined by Q-RT-PCR in RIP140^−/−^ brown or white adipocytes in the absence and presence of XCT790 (10 μM). n.d. not detected. For Q-RT-PCR, relative means ± SEM of biological triplicates are presented. *, *P* < .05, Student's *t* test for comparison of vehicle to XCT790.

RIP140 has been found, in WAT, to repress a set of genes normally associated with BAT. However, its role in brown adipocytes is less well defined. We generated conditionally immortalized preadipocyte cell lines from both BAT and WAT of mice heterozygous or null for the *NRIP1* gene. Heterozygous cells were selected for control because heterozygous mice are phenotypically similar regarding body weight (13), and brown fat gene expression was similar to that found in wild-type WAT and BAT (Supplemental Figure 1A). Oil Red O staining showed that all the cell lines differentiated efficiently following treatment with a standard differentiation cocktail (Figure 1A) in agreement with previous analysis showing that absence of RIP140 does not affect the ability of adipocytes to fully differentiate (8, 11). The mRNA level of genes highly expressed in white or brown adipocytes was assessed in the different cell lines following differentiation. The adipocyte marker Fabp4/aP2 was unaffected by the absence or presence of RIP140. Genes that are normally expressed at high levels in brown fat were elevated in the RIP140-null white adipocytes including Ucp1, Cidea, Cpt1b, Bmp8b (Figure 1B). It is noteworthy that RIP140 even repressed one of the key instigators of the brown adipocyte program Prdm16. Surprisingly, very few of the genes investigated were affected by the absence of RIP140 in brown adipocytes. Strikingly, Ptgds, which was elevated in the absence of RIP140 in white adipocytes (60-fold), was also increased in the RIP140^−/−^ brown adipocytes (30-fold) compared with RIP140-expressing cells. The only other genes increased in the absence of RIP140 in brown adipocytes were Cidea and Cpt1b and only around 2-fold compared to 200-fold and 7-fold, respectively, for these genes in white adipocytes. The minor changes in brown adipocyte gene expression correlate with the similar histologies and similar UCP1 and CIDEA staining of BAT from wild-type and RIP140-null mice (Supplemental Figure 2).

Although the basal levels of Ucp1 and PGC-1α were unaffected by the absence of RIP140 in brown adipocytes, the induction of these genes due to a 6-hour treatment with the selective β3-adrenergic ligand CL 316,234 resulted in significantly greater induction in cells lacking RIP140 (Figure 1C). The levels of the β3-adrenergic receptor were similar in both genotypes and down-regulated upon CL 316,234 treatment.

Ptgds showed the greatest increase in expression due to RIP140 ablation in brown adipocytes of all genes monitored. Because RIP140 functions to regulate NR activity we investigated whether ligands for PPARs affected Ptgds expression. The PPARγ and PPARα ligands rosiglitazone and GW7647 did not affect Ptgds expression (data not shown). Because we had previously found ERRα to be important for RIP140-dependent regulation of Ucp1 and Cidea (14, 30), we tested whether the ERRα inverse agonist XCT790 could affect Ptgds expression. We found that, in both white and brown adipocytes lacking RIP140, XCT790 potently repressed Ptgds mRNA expression (Figure 1D).

**Figure 2.**
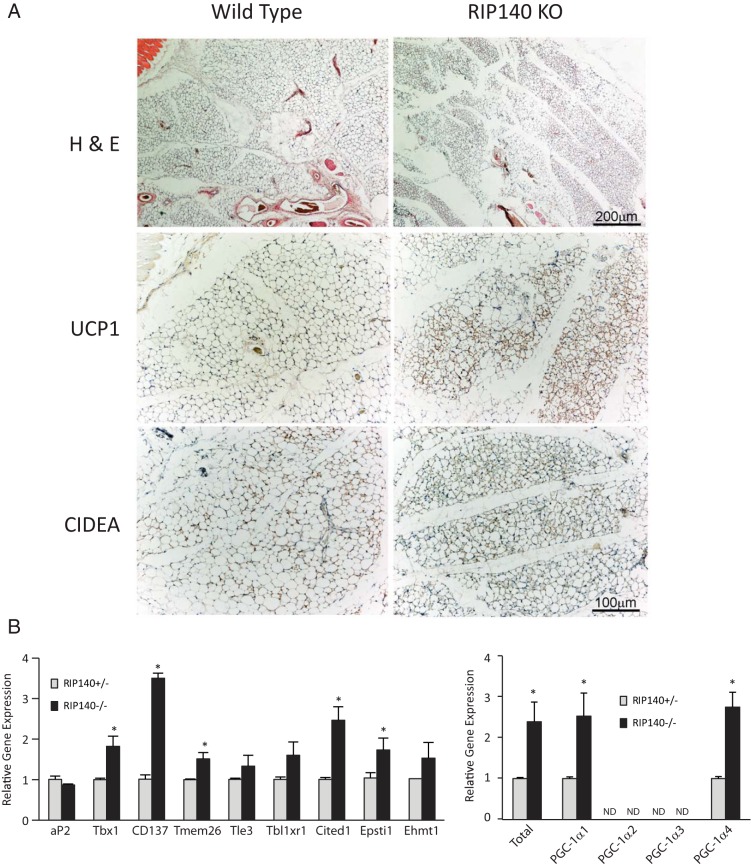

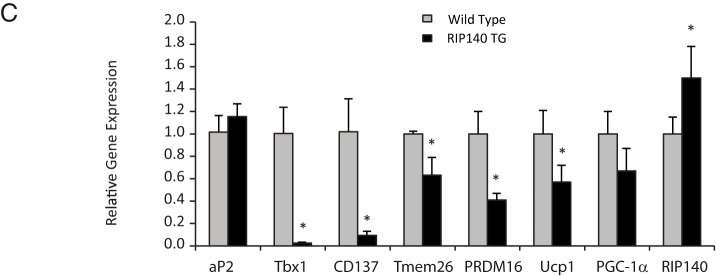
RIP140 suppresses the BRITE adipocyte program in white adipocytes. A, Hematoxylin and eosin (H & E) was performed on sc WAT from wild-type (n = 5) and RIP140^−/−^ (KO) mice (n = 6) to asses morphology (top row). The tissue in KO mice disclosed the appearance of a higher number of UCP1-positive multilocular adipocytes compared with wild type (middle row). The multilocular adipocytes were also positive for CIDEA, and the expression was increased in KO mice (lower row). Scale bar, 200 μm, applies to the top row, and 100 μm applies to the remaining 4 images. B, Gene expression was determined in undifferentiated sc white preadipocyte cell lines knockout or heterozygous for RIP140. Expression is presented relative to RIP140^+/−^ cells. *, *P* < .05 (Student's t test) for comparison of RIP140^+/−^ vs ^−/−^ cells, for individual genes. C, Gene expression was determined in sc WATs, wild type, or expressing a RIP140 transgene (TG). Expression is presented relative to wild type. For Q-RT-PCR, relative means ± SEM of biological triplicates are presented. *, *P* < .05 (Student's *t* test) for comparison of wild-type vs transgenic for individual genes.

Given that ablation of RIP140 resulted in the altered expression of many more genes in white compared with brown adipocytes and one of the affected genes was Prdm16, which is considered to be important in the early stages of brown adipocyte differentiation, we next focused on RIP140 and the BRITE program. Hematoxylin and eosin staining showed a predominance of adipocytes with multilocular morphology in RIP140 KO sc WAT unlike the unilocular appearance in wild-type tissue (Figure 2A). Furthermore, the brown adipocyte/BRITE markers UCP1 and CIDEA showed greater expression in the knockout tissue with most staining in the multilocular cells (Figure 2A) in agreement with mRNA expression (Supplemental Figure 1A). Next, we monitored the expression of the recently identified BRITE markers Tbx1, CD137, Tmem26, Cited1, and Epsti1 (31, 32) and found they were up-regulated in RIP140-null preadipocytes (Figure 2B). Tle3, Ehmt1, and Tbl1xr1 (33–35) did not show a statistically significant increase in expression in the null cells. Because PGC-1α is a key regulator of brown adipocyte/BRITE gene expression we monitored the levels of its different transcripts (36). Total PGC-1α, isoform 1, and isoform 4 were up-regulated in the absence of RIP140, whereas isoforms 2 and 3 were not detectable in either cell line (Figure 2B).

The genes Tbx1, CD137, and Tmem26 were down-regulated in the WAT of a mouse model in which a RIP140 transgene is overexpressed (Figure 2C) from a β-actin promoter (37). RIP140 mRNA and protein were increased in the transgenic mouse (Figure 2B and Supplemental Figure 1B). Prdm16 levels were also reduced, as was PGC-1α and Ucp1, in the presence of the RIP140 transgene. Taken together, these data indicate that RIP140 represses the BRITE gene expression program in WAT.

To investigate the action of RIP140 in white fat, we performed microarray analysis using RNA prepared from wild-type and RIP140-null WAT. We identified 736 probe sets to be significantly altered between the 2 genotypes, with 510 up-regulated and 226 down-regulated supporting a primary role as a corepressor in this tissue. To further characterize the genes controlled by RIP140, we compared the top 100 RIP140-repressed genes to gene lists generated from a separate microarray analysis of BAT, sc WAT, and mesenteric WAT from mice exposed to either 28°C or 6°C for 10 days. Of the top 100 genes repressed by RIP140, 65 were more highly expressed in BAT compared with the WAT depots with 56 genes being BAT-enriched compared with both sc and mesenteric WAT (Table 1). From the analysis we could identify a set of RIP140-regulated genes that were enriched in BAT compared with WAT and cold regulated. Essentially, the genes in this group are RIP140 targets in the BRITE program and include Fabp3, Otop1, Pank1, Lipa, ERRγ, Gyk, Kng1, PPARα, Pdk4, and Ptgds (Table 1).

**Table 1.** RIP140 Represses the Expression of Genes Associated With the BRITE Adipocyte Program

Gene Symbol	logFC	adj.P.Val	Cold-Induced (BAT)	Cold-Induced (SC)	BAT-Enriched (BAT vs *SC*)	BAT-Enriched (BAT vs *MES*)
Fabp3	−3.93	3.12E-03	+	+	+	+
Otop1	−2.83	5.27E-03	+	+	+	+
Pank1	−1.93	4.41E-03	+	+	+	+
Adrbk2	−1.79	2.40E-03	+	+	+	+
Kcnk3	−1.52	7.48E-04	+	+	+	+
NA (Affy ID: 1441765_at)	−1.47	1.53E-03	+	+	+	+
Lipa	−1.40	3.37E-05	+	+	+	+
9030617O03Rik	−1.35	3.47E-05	+	+	+	+
Esrrg	−1.15	8.99E-03	+	+	+	+
Gyk	−1.13	9.13E-04	+	+	+	+
9230104K21Rik	−1.13	2.26E-03	+	+	+	+
Igsf21	−1.08	4.26E-03	+	+	+	+
Mcart1	−1.00	1.96E-03	+	+	+	+
Kng1	−3.14	6.66E-07		+	+	+
Pdk4	−2.21	1.74E-03		+	+	+
Slc25a42	−2.15	2.22E-05		+	+	+
Ppara	−1.96	1.13E-03		+	+	+
Acaa2	−1.66	9.93E-04		+	+	+
Tmem37	−1.56	3.92E-03		+	+	+
Pdk2	−1.40	7.78E-04		+	+	+
Tob2	−1.38	1.38E-03		+	+	+
Atp1a2	−1.29	6.15E-05		+	+	+
Oplah	−1.18	4.12E-03		+	+	+
Ccs	−1.15	9.24E-05		+	+	+
Itpk1	−1.07	2.45E-03		+	+	+
Crls1	−1.07	5.27E-03		+	+	+
Ptgds	−2.58	1.03E-03	+		+	+
4930506M07Rik	−1.79	1.42E-04	+		+	+
1700040L02Rik	−1.71	1.26E-03	+		+	+
Cad	−1.49	5.05E-05	+		+	+
Rgnef	−1.04	1.35E-03	+		+	+
Cdh2	−1.81	5.85E-03			+	+
Clic5	−1.76	1.15E-03			+	+
Peg3	−1.64	5.85E-03			+	+
Prelp	−1.59	2.25E-03			+	+
Acss1	−1.46	4.42E-03			+	+
Khdrbs3	−1.43	4.75E-03			+	+
NA (Affy ID: 1457444_at)	−1.41	6.70E-04			+	+
St6galnac6	−1.37	3.55E-04			+	+
Prkag2	−1.29	2.44E-04			+	+
Copg2as2	−1.24	8.45E-03			+	+
Ttc28	−1.21	3.10E-04			+	+
Heyl	−1.21	6.96E-03			+	+
Nfic	−1.20	1.41E-04			+	+
NA (Affy ID: 1442102_at)	−1.18	2.26E-03			+	+
Flot2	−1.14	3.50E-05			+	+
Cgnl1	−1.09	2.25E-03			+	+
Ccbl1	−1.09	1.19E-04			+	+
Trim2	−1.08	9.67E-03			+	+
Ttc28	−1.05	5.06E-03			+	+
Gmnn	−1.04	3.45E-03			+	+
NA (Affy ID: 1439477_at)	−1.03	1.86E-03			+	+
Npc1	−1.03	4.35E-03			+	+
Tmem109	−1.02	4.88E-05			+	+
2810055F11Rik	−1.00	4.11E-04			+	+
Psme3	−1.00	1.15E-04			+	+
Ttpa	−1.58	3.01E-03	+			+
Rarres2	−1.37	1.91E-03	+		+	

Microarray analysis was performed on wild-type and RIP140 knockout WAT. Of the top 100 significantly RIP140-regulated genes, genes that were up-regulated in the absence of RIP140 were categorized as induced in interscapular BAT or sc WAT from a microarray analysis of tissue from mice exposed to 6°C vs 28°C. In addition, genes were categorized as BAT-enriched in BAT vs sc WAT or BAT vs mesenteric WAT, from mice maintained at 28°C.

**Figure 3.**
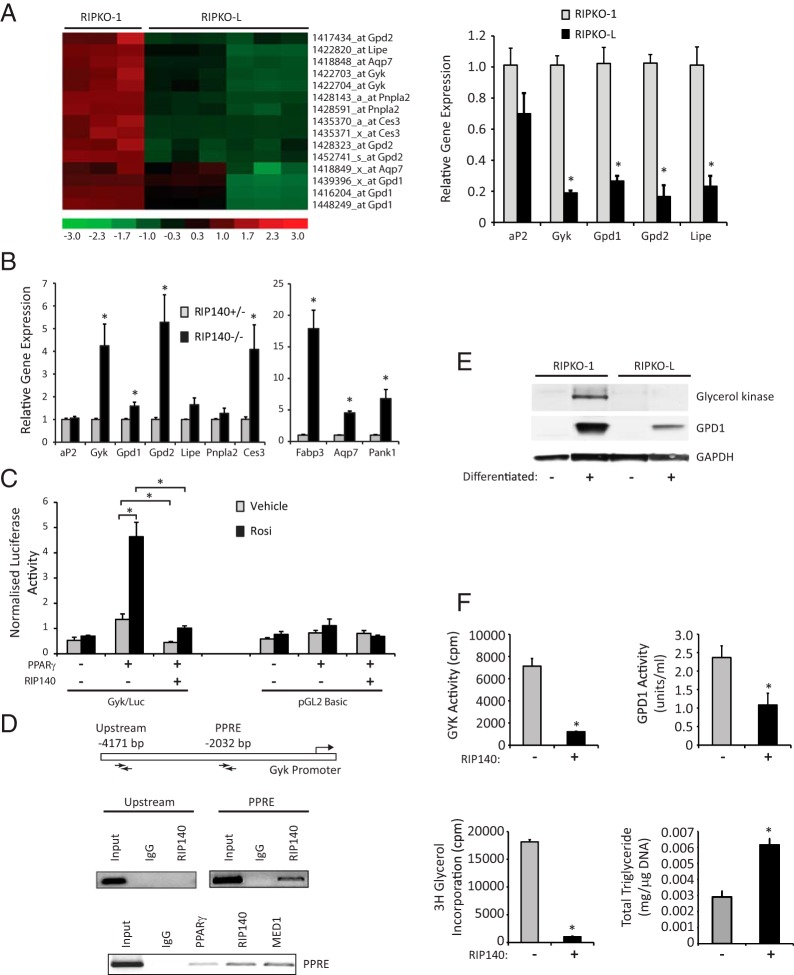
RIP140 suppresses genes that control a futile cycle of TAG breakdown and resynthesis. A, Cluster analysis of gene expression in differentiated RIPKO-1 and RIPKO-L cells as determined by Affymetrix microarray analysis. Gene expression for each sample is shown in columns; level of expression for each gene across samples, along with each gene name, is shown in rows. The scale relating level of gene expression with color is shown with −3 (bright green) being low and +3 (bright red) being high expression. Expression is relative to median levels of the 18 samples. Q-RT-PCR monitoring expression of aP2, Gyk, Gpd1, Gpd2, and Lipe after differentiation in RIPKO-1 and RIPKO-L cells (upper left panel). Data are expressed relative to expression in RIPKO-1 cells. *, *P* < .05 (Student's t test) for comparison of RIPKO-1 vs RIPKO-L for individual genes. B, Expression of the indicated genes was determined in RIP140^−/−^ and RIP140^+/−^ differentiated sc white adipocytes (lower panel). Data are expressed relative to expression in RIP140^−/−^ cells. *, *P* < .05 (Student's *t* test) for comparison of RIP140^+/−^ vs ^−/−^ cells for expression of individual genes. C, Transient transfection of 293 cells with Gyk promoter reporter construct, or pGL2 Basic, in the absence and presence of PPARγ and RIP140. Cells were treated with vehicle or 1 μM rosiglitazone (Rosi). *, *P* < .05 (ANOVA) with Bonferroni's correction for comparison of normalized luciferase activities in response to PPARγ, RIP140, and ligand treatment, for individual reporter constructs. D, Schematic representation of the *Gyk* gene indicating the position of the primers used for ChIP assay at the –2032-bp enhancer element that encompasses a PPAR response element and an unrelated control region at −4171 bp. ChIP assay using specific antibodies for RIP140, PPARγ, and Med1 in 3T3–L1 adipocytes for the *Gyk* enhancer and upstream control element. E, Western blots of whole-protein extracts from RIPKO-1 and +RIPKO-L preadipocytes and adipocytes probed with antibodies against GYK and GPD1. F, Relative GYK enzymatic activity in vitro determined as [^3^H]glycerol phosphorylated to [^3^H]G3P in the presence of GYK isolated from RIPKO-1 and RIPKO-L adipocytes. Relative enzymatic activity of GPD1 in vitro determined as decrease in NADH measured by absorbance in the presence of substrate and isolated GPD1 from RIPKO-1 and RIPKO-L adipocytes. The incorporation of [^3^H]glycerol in isolated fat fractions from RIPKO-1, and RIPKO-L. Total cellular triglyceride levels were determined for RIPKO-1 and RIPKO-L adipocytes. For Q-RT-PCR, reporter assays, and functional assays, means ± SEM of biological triplicates are presented. *, *P* < .05 (Student's *t* test) for comparison of RIPKO-1 vs RIPKO-L.

It was surprising that the gene Gyk was repressed by RIP140 because it is required for TAG synthesis and previous studies indicated that, in adipocytes, RIP140 was a repressor of genes associated with catabolic rather than anabolic processes. A clustering analysis of genes involved in glycerol metabolism that are specifically up-regulated in RIP140-null adipocytes is presented in Figure 3A. These include the glycerol phosphorylation enzyme Gyk, the lipases Lipe, Pnpla2 and Ces3, the glycerol-specific membrane transporter aquaporin 7 (Aqp7), and the glycerol-3-phosphate dehydrogenase enzymes 1 and 2 (Gpd1 and Gpd2). Expression of key rate-limiting enzymes of the TAG recycling mechanism on RNA from independent adipocyte cells null for RIP140 (RIPKO-1) and with lentiviral-mediated stable RIP140 expression (RIPKO-L) was analyzed by Q-RT-PCR (Figure 3A). Gyk showed 5-fold higher expression levels in RIP140-null adipocytes, Lipe showed a 4.5-fold increase, whereas Gpd1 and Gpd2 showed 3.3-fold and 5.6-fold increases, respectively, confirming the microarray data. A similar pattern was observed in sc white preadipocytes in which Gyk, Gpd1, and Gpd2 were significantly up-regulated in the absence of RIP140. Fabp3, Pank1, Aqp7, and the lipase Ces3 were also increased in adipocytes knockout for RIP140 (Figure 3B).

RIP140 repressed Gyk promoter-driven reporter gene activity when cotransfected with PPARγ (Figure 3C). Chromatin immunoprecipitation showed that RIP140 was specifically bound to a PPAR response element (PPRE) in the Gyk promoter and not detected in an upstream control region (Figure 3D). Furthermore, PPARγ and the coregulator MED1 were also found to be associated with the Gyk PPRE (Figure 3D). Western blotting revealed that both GYK and GPD1 protein were expressed in adipocytes and not preadipocytes, and levels were reduced when RIP140 was expressed (Figure 3E). Finally, functional assays were undertaken to determine the impact of RIP140 on glycerol metabolism. Glycerol kinase activity, glycerol-3-phosphate dehydrogenase activity, and [^3^H]glycerol incorporation into TAG were all reduced in RIP140-expressing compared with null adipocyte (Figure 3F). Furthermore, the total cellular TAG levels were lower in the RIPKO cells (Figure 3F). Thus, RIP140 inhibits the synthesis of TAG due to repression of key genes required for the metabolism of glycerol.

## Discussion

In recent years there has been a renaissance in brown fat research, given its identification in adult humans (5, 38). BAT exists in a defined interscapular sc depot in mice although brown adipocytes also exist within white-fat depots. These BRITE cells are detected upon exposure to stimuli such as cold. In adult humans, the detectable regions of brown fat appear to be similar to the BRITE cells (32). We determined RIP140 to be a regulator of the BRITE program, with increased abundance of multilocular adipocytes and UCP1 and CIDEA-stained cells in sc WAT of RIP140 KO. This was in contrast to the BAT, which showed similar histology and staining when compared with wild type. In white adipocytes mRNA expression of late-expressed markers such as Ucp1 as well as early markers including Prdm16 (39), and Tbx1, CD137, Tmem26 (31), Cited1, and Epsti1 (32) were elevated in the absence of RIP140. Thus, RIP140 appears to have 2 distinct roles in adipocytes: 1) to repress regulators of the BRITE program and 2) to repress genes expressed in differentiated BRITE cells such as Ucp1 and Cidea (11).

A number of factors that switch white adipocytes to express brown adipocyte genes have been identified including PGC-1α (40), PRDM16 (41), and the myokine irisin (42). The transcription factors and coregulators that facilitate “browning” of white adipocytes have mostly been studied in cell-based systems. RIP140 is a significant player in this process because its absence in vivo, in white fat, results in the induction of “brown fat” gene expression. Unlike the other regulators of the white-to-brown switch RIP140 has a relatively minor role in brown adipocytes. Its primary function in appears to be suppression of the BRITE phenotype in white adipocytes. This may involve stoichiometric inhibition of PGC-1α-dependent gene expression through promoter-bound NRs or a direct interaction between these 2 coregulators (14). The ratio of PGC-1α to RIP140 gene expression differs between white and brown fat depots. The higher ratio is found in mouse interscapular BAT compared with visceral and sc WAT as well as human BAT compared with WAT (43). In addition, RIP140 may directly modulate expression of PGC-1α. We found PGC-1α isoforms 1 and 4, as well as total PGC-1α, were repressed by RIP140, and isoforms 2 and 3 were not detected in brown adipocytes. It is noteworthy that although RIP140 has a minor transcriptional role in brown adipocytes, it may have other important roles, for example as a regulator of glucose trafficking through interaction with AS160, which reduces the level of AKT-dependent phosphorylation impeding its inactivation, thus maintaining its inhibition of GLUT4 trafficking (44).

The gene Ptgds, encoding for lipocalin prostaglandin D synthase, stood out as induced following RIP140 ablation in both brown and white adipocytes. We recently reported that it controls fuel utilization in BAT because its absence resulted in increased glucose utilization (45). In the current study, we addressed the NRs that may determine the expression of Ptgds through coregulators such as PGC-1 and RIP140. Although we found that Ptgds levels were not affected by PPAR agonists, we did find that ERRα was required for its expression. Because ERRα, coregulated by PGC-1α and RIP140, is also important for Ucp1 and Cidea expression (14, 46), the interplay between these 3 transcriptional regulators seems to be a mechanism common to the regulation of many brown adipocyte genes.

**Figure 4.**
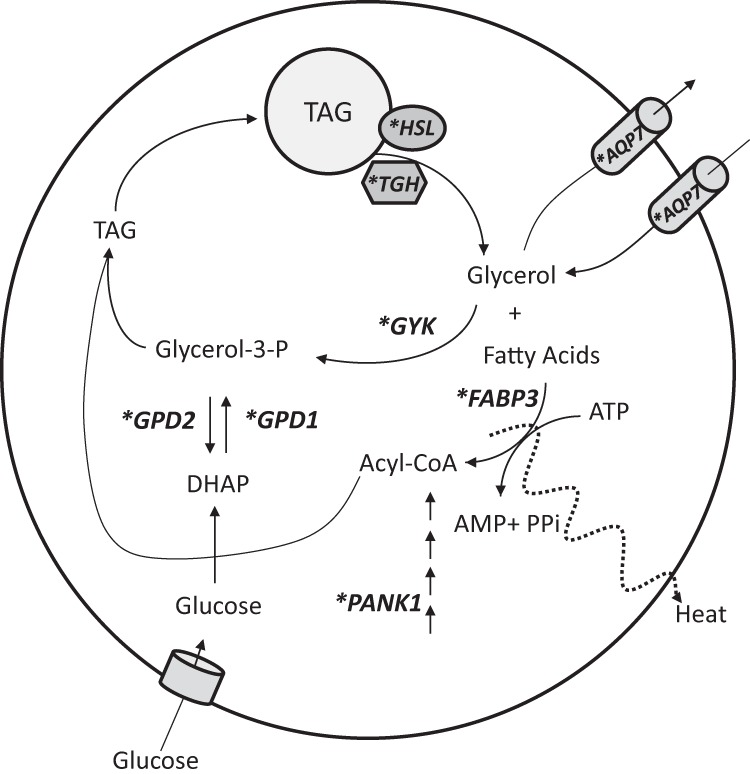
Schematic showing the TAG futile cycle under the control of RIP140. A futile metabolic cycle of glycerol recycling is activated in RIP140-null adipocytes. Asterisk indicates RIP140-regulated genes. The induction of GYK and GPD1 in the absence of RIP140 allows for an intracellular recycling of TAG, with breakdown followed by resynthesis that utilizes energy and shifts the metabolic profile of adipocytes toward energy expenditure. Lipolysis is facilitated by hormone-sensitive lipase (HSL) and Carboxylesterase 3 (Ces3)/TAG hydrolase (TGH). Pantothenate kinase 1 (PANK1) is a key regulatory enzyme in the biosynthesis of CoA, FABP3 is a fatty acid transporter, and AQP7 is a glycerol transporter.

Most of the genes that are elevated in WAT due to RIP140 ablation are more highly expressed in brown vs white fat. We have identified RIP140-regulated genes that are cold induced and associated with higher expression in brown fat. This stringent data analysis highlights BRITE genes that may be direct targets for RIP140 and include Fabp3, Otop1, Pank1, Adrbk2, Kcnk3, Lipa, Errg, Gyk, Igsf21, and Mcart1. Of these genes Gyk and Pank1 are important in a TAG synthesis. This is somewhat surprising, given that RIP140, in adipocytes, has been linked with suppression of energy utilization rather than anabolic pathways. Furthermore, a clustering analysis of RIP140-regulated genes in differentiated adipocytes revealed additional up-regulated genes associated with both TAG synthesis and breakdown. A futile cycle has been reported in response to thiazolidinediones and PGC-1α and PPARα (28, 47) as well as due to cold stress in humans (48). This cycle of TAG hydrolysis and resynthesis requires substantial amounts of ATP, because it is used for the activation of free fatty acids by acyl-coenzyme A (CoA) synthetases and for the phosphorylation of glycerol by GYK (Figure. 4). The expression of GYK in adipocytes allows for any glycerol that arises from increased lipolysis to be phosphorylated to glycerol-3-phosphate and recombined with free fatty acid to form TAG. Gyk expression is primarily regulated by PPARγ, and RIP140 represses the PPARγ-dependent activation of the Gyk promoter and binds directly to a previously reported PPRE on its promoter. Moreover, the protein and relative enzymatic activity of GYK is strikingly increased in RIP140^−/−^ adipocytes, and the resulting phosphorylated glycerol is incorporated into fat, confirming a futile cycle of glycerol recycling.

Lipolysis could be elevated in the absence of RIP140 due to the increased expression of the adipocyte lipases hormone-sensitive lipase and Ces3/ triacylglycerol (TAG) hydrolase (49) (Figure 4). The adipose glycerol transporter Aqp7 was induced in RIP140^−/−^ adipose tissue and may be an important component of the futile cycle. However, exactly how it would contribute to this cycle is open to question because it is considered primarily an exporter of glycerol. One possibility is that AQP7 functions as a bidirectional glycerol pore that prevents the net export of glycerol to enable sufficient glycerol levels for TAG synthesis. This is supported by studies in Aqp7 knockout adipocytes in which [^14^C]glycerol uptake was reduced by 3-fold compared with wild- type cells (50, 51). Furthermore, in the presence of elevated Aqp7, in response to pioglitazone or rosiglitazone in rodents, futile cycling of TAG is maintained without increased release of glycerol to the bloodstream (52, 53). It is likely that RIP140 functions to repress Aqp7 expression via an interaction with a PPAR because the Aqp7 promoter is regulated via a PPAR response element (52). PPARs are important targets for RIP140 in adipocytes (46), and pantothenate kinase (Pank1) is a PPARα-regulated gene (54) that we also found to be repressed by RIP140. It catalyzes the first committed step and controls the overall rate of CoA biosynthesis (55) and would facilitate the high levels of acyl-CoA for TAG synthesis or β-oxidation, that would occur in activated brown or BRITE adipocytes.

The expression of cytosolic and mitochondrial dehydrogenases Gpd1 and Gpd2 is also up-regulated in the absence of RIP140. The activity and protein levels of GPD1, which converts glucose and pyruvate-derived dihydroxyacetone phosphate to glucose-3-phosphate, are increased in RIP140-null adipocytes although to a lesser extent relative to GYK activity. The 2 glycerophosphate dehydrogenases form the glycerol phosphate shuttle, which provides for mitochondrial oxidation of reduced nicotinamide adenine dinucleotide produced in the cytoplasm (56). The elevated expression of these enzymes could reflect increased activity of the shuttle to accommodate increased glycolysis.

The futile cycle may be an important contributor to Ucp1-independent thermogenesis during exposure to cold. We found that Gyk and Aqp7, in addition to being more highly expressed in brown compared with white depots, were increased in WAT depots following cold acclimatization (57). This cycle could provide unilocular WAT cells with a thermogenic potential, which would be completed with the increase in mitochondria number and UCP1 levels acquired during their transdifferentiation (19) to BRITE adipocytes. The viability of Ucp1-null mice when exposed to the cold (4 or 5°C) reveals the presence of alternative mechanisms, independent of UCP1, to maintain body temperature (58–60). In fact, Ucp1-knockout mice are still capable of exhibiting 50% of the wild-type increase in maximal cold-induced heat production. A futile cycle of TAG hydrolysis and resynthesis in the adipose tissues of these mice could represent an important mechanism for heat generation and their survival in the cold in the absence of UCP1.

The inappropriate expression of the BRITE phenotype in WAT would drive excessive energy utilization and therefore be a survival disadvantage. RIP140 can be ascribed to be a “thrifty gene” fulfilling this role by suppression of aspects of the brown/BRITE adipocyte phenotype. The RIP140-knockout mouse provides a novel genetic model for BRITE adipocytes in white fat in the absence of stimulus such as cold exposure. RIP140 represses genes that 1) mediate a futile cycle of TAG breakdown and resynthesis, 2) uncouple respiration and 3) are early markers of the brown/BRITE program. Thus, RIP140 represents an important target for strategies to increase the number of brown adipocytes within white fat depots.

## Additional material

Supplementary data supplied by authors.
