# Supplementary material for: RIP140 Represses the “Brown-in-White” Adipocyte Program Including a Futile Cycle of Triacyclglycerol Breakdown and Synthesis
Source: Mol Endocrinol. 2014 Jan 30;28(3):344–56. doi: 10.1210/me.2013-1254 (PMC4207910; doi:10.1210/me.2013-1254)
Supplement: Supplementary file 1 [file me-13-1254.pdf]

Supplementary Figure 1

A) **Ucp1 and Cidea gene expression in white and brown adipose tissues.** Gene expression was determined in subcutaneous white adipose tissue (WAT) and interscapular brown adipose tissue (BAT). Tissues were collected from wild type (WT), RIP140-/+ and RIP140-/- mice. Expression is presented relative to WT WAT. *p<0.05 Student's t test in comparision to WT for each tissue.

B) **RIP140 protein expression in RIP140-transgenic white adipose tissue.** Protein extracts from subcutaneous white adipose tissue from wild type (WT) and RIP140 transgenic (TG) mice were separate by SDS-PAGE, transferred to PVDF membrane and exposed to anti-RIP140 (6D7) or anti-b-actin antibody (Abcam). Bands were visualised with ECL2 (Thermo Scientific Pierce).

Supplementary Figure 2

Histology of RIP140 Knockout Brown Adipose Tissue. Representative images of hematoxylin and eosin (H&E) staining performed on brown adipose tissue (BAT) sections from wild type and RIP140 knockout (KO) mice (top row). UCP1 (middle row) and CIDEA staining (lower row) did not showed clear differences in BAT obtained from wild type in comparison to KO mice. Scale bar 200µm apply to H & E and 100µm to middle and lower panels.

A

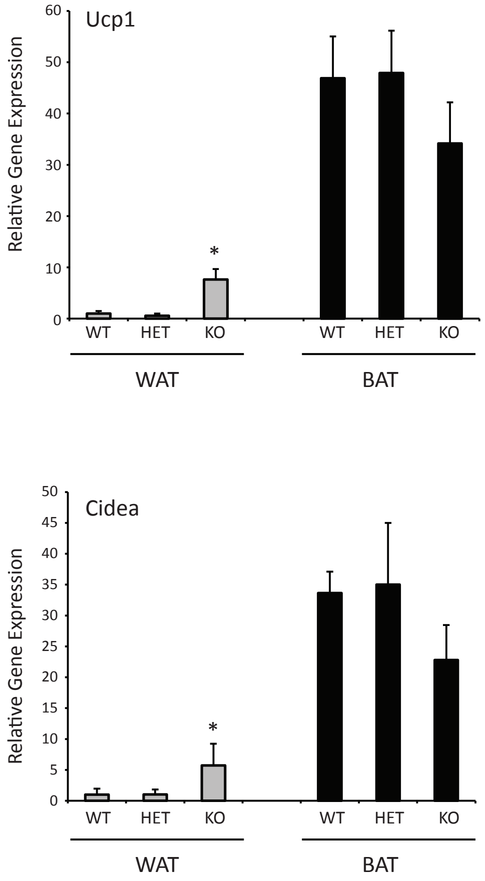
Ucp1
Relative Gene Expression
0
10
20
30
40
50
60
*
WT
HET
KO
WT
HET
KO
WAT
BAT
Cidea
Relative Gene Expression
0
5
10
15
20
25
30
35
40
45
50
*
WT
HET
KO
WT
HET
KO
WAT
BAT

B

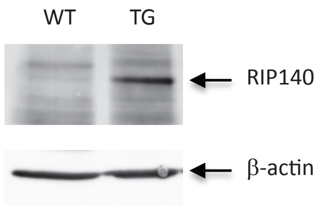
WT
TG
RIP140
β-actin

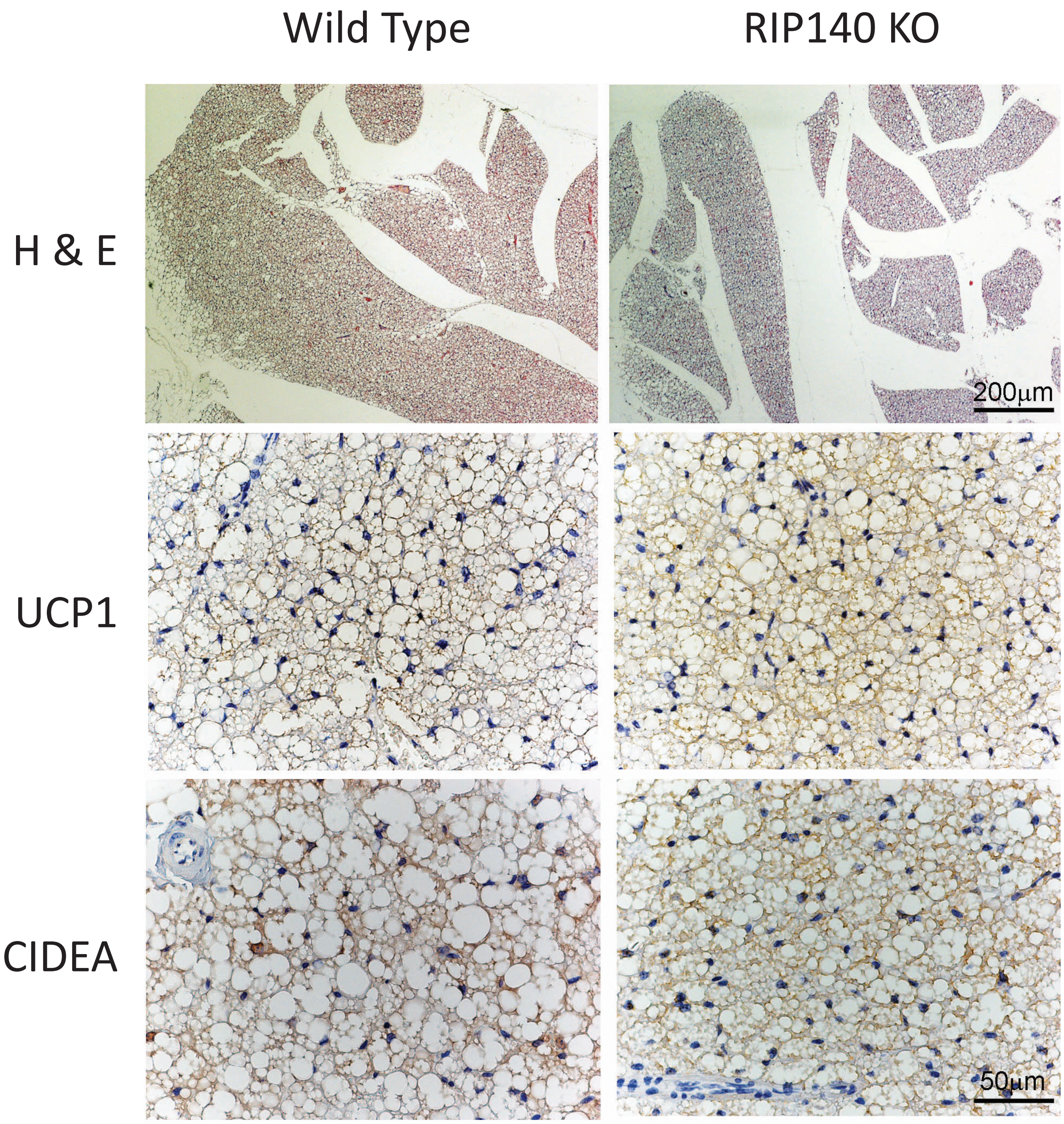

Wild Type
RIP140 KO
H & E
UCP1
CIDEA
200μm
50μm

Supplementary Table 1. Sequences of primers used for Q-RT-PCR

	Forward Primer (5' to 3')	Reverse Primer (5' to 3')
**ACC1**	TGTCCACCCAAGCATTTCTTC	CATCCAACACCAGTTCAGTATACGT
**ACC2**	ACTTTGACCTGACCGCTGTG	CTGAGTGCCGGATAATGGC
**aP2**	ACACCGAGATTTCCTTCAAACTG	CCATCTAGGGTTATGATGCTCTTCA
**Bmp8b**	ACCCCGCAGCCTCACTTGGACC	TGGCTCCTGGTAGCCCAGGGT
**Cd137**	CGTGCAGAACTCCTGTGATAAC	GTCCACCTATGCTGGAGAAGG
**Cidea**	AAGCTTCAAGGCCGTGTTA	CTGTAGCTGTGCCCTGGTTA
**Cpt1b**	ACCACATCCGCCAAGCA	TTCCTCAGCTGTCTGTCTTGGA
**Elovl6**	TGTGCTCAGCAAAGCACCCGA	TGTGGTGGTACCAGTGCAGGA
**Ehmt1**	GGCACCTTTGTCTGCGAATAC	AGAACCGAGCGTCAATGCAG
**FAS**	TGCGGAAACTTCAGGAAATGT	AGAGACGTGTCACTCCTGGACTT
**Gpd1**	GCAAGATCTGTGACCAGCTCAA	GCCCTCGTCTACCCCCTTAA
**Gpd2**	CCACGGTGGTGTGCGATAC	AGGGCTTCTTTCACCATCCTATACT
**Gpr120**	ACATTGGATTGGCCCAACCGCA	TCCGCGATGCTTTCGTGATCTGT
**Gyk**	TCCAACATCAAAGCCATTGGT	AGAGGCTCTCCGGTGACCTT
**HSL**	CGAGACAGGCCTCAGTGTGA	TCTGGGTCTATGGCGAATCG
**L19**	GGAAAAAGAAGGTCTGGTTGGA	TGATCTGCTGACGGGAGTTG
**LETM1**	CCCGGGCCATGTACCTCCCA	CCTGGGCCTCCTTTGCCACA
**Pdk4**	TGACTCAAAGACGGGAAACC	ACTGGTCGCAGAGCATCTTT
**PGC-1a**	CCCTGCCATTGTTAAGACC	TGCTGCTGTTCCTGTTTTC
**Plin5**	TCCTGCCCGTCAAAGGGATCTGA	GGACATTCTGCTGTGTGGCGCT
**PPARa**	GCGTACGGCAATGGCTTTAT	GAACGGCTTCCTCAGGTTCTT
**PPARγ**	GTGCCAGTTTCGATCCGTAGA	GGCCAGCATCGTGTAGATGA
**Prdm16**	CAGCACGGTGTACCCAT	CCGGTGCATCCGGTTGTC
**Ptgds**	GGCTCCTGGACACTACACCTACA	ATAGTTGGCCTCCACCACTGA
**Rgs7**	CCAGTGTGTTCTCCGGGTAGA	ATGGGCAGCCATTAGCGTTCCC
**RIKEN9**	TTGGCCCTGGAGGCAAGGGG	TGGGAACCCTGAAGGACCCCA
**RIP140**	TCAAAAGCCACACCATACCC	TCGTCTCCACTGCTGTCATC
**SCD1**	GTACGACTGGGCCCGAGAT	TGAGGGTCGGCGTGTGT
**Slc27a2**	TGTATGGCGTGCCTGTGCCA	GAGGCCTCGCGTAACTGGGC
**ß3AR**	ATCGTGTCCGCTGCCGT	ATCTGCCCCTACACGCCAC
**Ucp1**	ACTGCCACACCTCCAGTCATT	CTTTGCCTCACTCAGGATTGG
**Tmem26**	ACCCTGTCATCCCACAGAG	TGTTTGGTGGAGTCCTAAGGTC
**Tbx1**	GGCAGGCAGACGAATGTTC	TTGTCATCTACGGGCACAAAG
